# Lavandula Reduces Heart Injury via Attenuating Tumor Necrosis Factor-Alpha and Oxidative Stress in A Rat Model of Infarct-Like Myocardial Injury 

**DOI:** 10.22074/cellj.2016.4148

**Published:** 2016-12-21

**Authors:** Jafar Sadeghzadeh, Abedin Vakili, Ahmad Reza Bandegi, Hamid Reza Sameni, Mahdi Zahedi khorasani, Mohsen Darabian

**Affiliations:** 1 Research Center and Department of Physiology, Faculty of Medicine, Semnan University of Medical Sciences, Semnan, Iran; 2 Department of Biochemistry, Faculty of Medicine, Semnan University of Medical Sciences, Semnan, Iran; 3 Research Center of Nervous System Stem Cells, Department of Anatomy, Faculty of Medicine, Semnan University of Medical Sciences, Semnan, Iran; 4 Department of Cardiology, Faculty of Medicine, Semnan University of Medical Sciences, Semnan, Iran

**Keywords:** Myocardial Infarction, TNF-α, Isoproterenol, Oxidative Stress, Rat

## Abstract

**Objective:**

Lavender is used in herbal medicine for different therapeutic purposes. Nonetheless, potential therapeutic effects of this plant in ischemic heart disease and its possible mechanisms remain to be investigated.

**Materials and Methods:**

In this experimental study, lavender oil at doses of 200, 400 or 800 mg/kg was administered through gastric gavage for 14 days before infarct-like myocardial injury (MI). The carotid artery and left ventricle were cannulated to record arterial blood pressure (BP) and cardiac function. At the end of experiment, the heart was removed and histopathological alteration, oxidative stress biomarkers as well as tumor necrosis factor-alpha (TNF-α) level were evaluated.

**Results:**

Induction of M.I caused cardiac dysfunction, increased levels of lipid peroxidation, TNF-α and troponin I in heart tissue (P<0.001). Pretreatment with lavender oil at doses of 200 and 400 mg/kg significantly reduced myocardial injury, troponin I and TNF-α. In addition, it improved cardiac function and antioxidant enzyme activity (P<0.01).

**Conclusion:**

Our finding showed that lavender oil has cardioprotective effect through inhibiting oxidative stress and inflammatory pathway in the rat model with infarct-like MI. We suggest that lavender oil may be helpful in prevention or attenuation of heart injury in patients with high risk of myocardial infarction and/or ischemic heart disease.

## Introduction

Cardiovascular diseases are becoming the main cause of mortality and morbidity around the most countries of world (1). Myocardial infarction is the most common form of heart disease. Today, traditional medicine has been more accepted by the people and modern medicine due to better understanding of the mechanisms and the impact on health and quality of life (2, 3). In recent decades, therapeutic effect of antioxidants in prevention of cardiovascular disease has been considered (4, 5). Epidemiological studies have shown that vegetables, fresh fruit or plants with rich antioxidant substances are useful in prevention of cardiovascular disease (6).

Lavender (*Lavandula angustifolia*), a member of the Labiatae family, is used for a variety of cosmetic and therapeutic purposes in herbal medicine (7, 8). Inhalation of essential oils of lavender reduced cholesterol plaques in atherosclerotic disease in rabbits, but showed no effect on serum cholesterol levels (9). Lavander showed a hypolipidemic effect in rats (10). In addition, lavender aromatherapy has displayed vasodilatory effects and enhanced coronary blood flow in human (11). Extract of lavender flower protected isolated rat hearts against ischemic reperfusion (IR) injury (12). In our recent study, lavender oil showed neuroprotective activity and antioxidant properties in an experimental model of stroke (13). In a very recent study, treatment with essential oil of lavender after MI reduced ischemic injury in rats (14). To the best of our knowledge, effects of pretreatment with lavender on myocardial ischemic injury have not been studied yet. This study aimed to investigate the effects of different doses of lavender oil and its possible mechanism in the prevention and/ or attenuation of heart damage in a rat model of infarct-like myocardial injury.

## Materials and Methods

### Animals and drugs 

In this experimental study, 65 wistar male rats (250 ± 50 g) were provided by breeding colony at Semnan University of Medical Sciences (SUMS), Semnan, Iran. Animals were kept in standard cages with free access to food and water, and all experiments were performed in accordance with the Research Ethics Committee and the national guidelines for conducting animal studies. Extract of lavender oil (*Lavandula angustifolia*) and isoproterenol (ISO) hydrochloride were obtained from Sigma company (Germany), oxidative markers from Biorexfars Company (UK) and thiopental sodium from Kwality Pharmaceuticals Pvt. Ltd. (India).

### Experimental protocol and design

The 65 animals were divided randomly into eight groups. The first group (n=8) was of normal control rats. In the second, third and fourth groups (n=7 each), normal rats received lavender oil at doses of 200, 400, and 800 mg/kg, respectively, by oral gavage daily for 14 days. In the fifth group (n=9), rats received ISO (85 mg/kg) as control. In the sixth group (n=10), rats were pretreated with lavender oil (200 mg/kg) and then acute MI was induced by ISO. In the seventh group (n=9), rats were pretreated with lavender oil (400 mg/ kg) and then acute MI was induced. The eighth group rats (n=8) pretreated with lavender oil (800 mg/kg) and then acute M.I was induced. Lavender oil (Sigma, UK) was given daily for a period of 14 days via oral gavage.

Twelve hours after the second dose of ISO injection, all rats were anesthetized with thiopental sodium (80 mg/kg, IP) and subsequently the femoral artery and the left ventricle of heart were cannulated for recording blood pressure (BP). In addition, hemodynamic parameters, cardiac function and blood samples were collected for troponin I measurement. Finally, two samples of left ventricular apex were removed immediately for measurement of biochemical and histological parameters. A known weight of the heart tissue was homogenized in 5.0 ml of 0.1 M Tris-HCl buffer (pH=7.4) solution. The homogenate was centrifuged and the supernatant was used to determine various biochemical parameters.

### Induction of infarct-like myocardial injury

Acute infarct-like myocardial injury (MI) was induced with subcutaneous injection of ISO (85 mg/kg) at intervals of 24 hours for two consecutive days. ISO produces an infarct-like myocardial lesion, cardiac dysfunction and other toxic manifestation in the rats, similar to acute myocardial infarction in humans.

### Measurement of cardiac troponin I

24 hours after induction of MI and at the end of experiment, 2 ml blood sample was collected from the carotid artery to measure cardiac troponin I (cTnI) levels. The levels of troponin I in serum were evaluated using a standard kit by enzyme-linked immunosorbent assay (Hangzhou Eastbiopharm Co., Ltd, China).

### Histopathological measurement

At the end of experiment, under deep anesthesia, the left ventricular cardiac apex was rapidly isolated and fixed in 10% buffered formalin solution. After fixation, the heart tissue was processed by embedding in paraffin, sectioned at 5 μm, and stained with hematoxylin and eosin (H&E) for histopathological examination under the light microscope (×200).

The slides were assessed for myonecrosis, inflammatory cell infiltration and edema. A minimum of 10 fields for each slide was examined and graded for severity of changes as follows. Grade 1 (): absence of inflammation, edema and necrosis, grade 2 (+): focal areas of inflammation, edema and necrosis, grade 3 (++): patchy areas of inflammation, edema, and necrosis, grade 4 (+++): confluent areas of inflammation, edema and necrosis, grade 5 (++++): massive areas of inflammation, edema and necrosis (15). The examiner was blind on the animal experimental groups’ information.

### Measurement of hemodynamic and cardiac parameters

20 hours after the second dose of ISO injection and under sodium thiopental anesthesia, a polyethylene cannula (PE-50) was inserted into the right common carotid artery for recording heart rate and arterial BP (Powerlab system, AD Instruments, Australia). To evaluate the cardiac left ventricular function, a polyethylene cannula was gently advanced into the left ventricular lumen for measurement of left ventricular systolic pressure (LVSP), left ventricular end-diastolic pressure (LVEDP), maximum rate of left ventricular (LV) pressure increase (LV dp/dt maximum, contraction velocity), and maximum rate of LV pressure decline (LV dP/dt minimum, relaxation velocity). These parameters were continuously monitored and recorded using a Powerlab system (AD Instruments, Australia).

### Oxidative stress biomarkers and tumor necrosis factor-alpha assay

The supernatant of heart tissues were used for biochemical analyses. Total protein in the heart homogenate was determined by the Bradford method (16). The Malondialdehyde (MDA) content, as a lipid peroxidation index, or thiobarbituric acid reactive substances (TBARS) were measured as described previously (17). Superoxide dismutase (SOD) and glutathione peroxidase (GPx) activities were measured using a commercial kit (Biorexfars Company, UK) and according to the manufacturer’s protocol. The colorimetric ferric reducing/antioxidant power (FRAP) assay was used for measuring total antioxidant capacity. Tumor necrosis factor-alpha (TNF-α) were measured using an enzyme-linked immunosorbent assay (ELISA) method in heart tissue using rat TNF alpha ELISA Kit (Biorbyt, United Kingdom). TNF-α levels were expressed as pg/mg protein.

### Statistical analysis

Statistical analysis was performed by two- way analysis of variance (ANOVA) followed by Tukey’s test using the GB-STAT software package version 5. Data are expressed as mean ± SEM. P<0.05 were considered significant.

## Results

### Effect of pretreatment with various doses of lavender oil on left ventricular function and hemodynamic parameters

**Fig.1:**
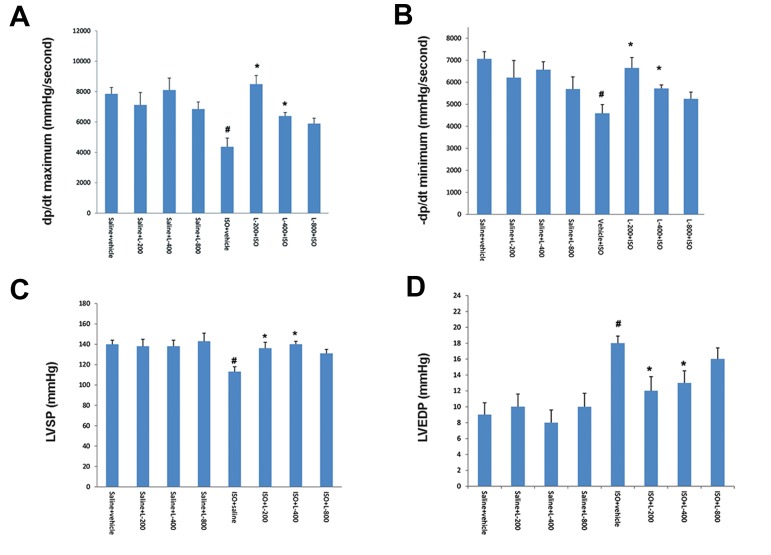
Effect of pretreatment with lavender oil (L) on left ventricular function. A. Maximum rate of left ventricular (LV) pressure increase (+LV dp/ dt maximum, contraction velocity), B. Maximum rate of LV pressure decline (LV dP/dt minimum-relaxation velocity), C. Left ventricular systolic pressure (LVSP), and D. Left ventricular end-diastolic pressure (LVEDP) in normal rat (saline+vehicle, saline+L-200, saline+L-400 and saline+L-800) and isoproterenol (ISO) groups including: ISO+vehicle, ISO+L-200, ISO+L-400 and ISO+L-800 mg/kg. Values are mean ± SEM. #; P<0.01 from respective saline+vehicle value and *; P<0.01 as compared to ISO+vehicle group.

Induction of acute MI by ISO resulted in significant (P≤0.01) left ventricular dysfunction, as shown by an increase in LVEDP, and diminution in LV dp/ dt maximum, LV dp/dt minimum, LVSP, and BP relative to normal control (saline) rats. Pretreatment with lavender oil at doses of 200 and 400 mg/ kg significantly (P≤0.05) improved all of these parameters in comparison with the ISO-treated group (Figes.1A-D, 2). Heart rate was not significantly changed (P>0.05) in ISO-treated rats in comparison with other groups. The rise in LVEDP in the ISO treated rats is an index of left ventricular dysfunction, which was significantly (P<0.05) attenuated by lavender oil pretreatment (Fig.1D).

### Effect of pretreatment with various doses of lavender oil on histopathological changes

The results of histopathological assessment under the light microscope in normal and lavender oil-treated groups are shown in Table 1. Histopathological examination of the treated normal rat with or without lavender oil showed normal muscle fibers with no pathological change (Fig.3).

ISO treatment induced severe myocardial necrosis with edema, leukocyte infiltration and splitting myofibrils cardiac (++++, grade 5). Pretreatment with lavender oil (200 and 400 mg/ kg) considerably reduced myocardial necrosis edema and leukocyte infiltration (+, grade 2), indicating near-normal structure of myofibril striations (Table 1, Fig.3).

**Fig.2:**
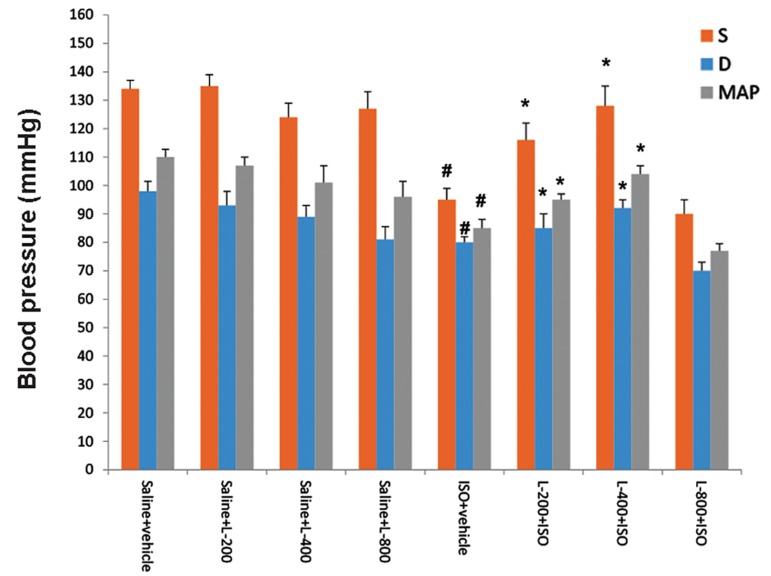
Effect of pretreatment with lavender oil (L) on systolic (S) and diastolic (D) and mean arterial blood pressure (MAP) in normal rat (saline+vehicle, saline+L-200, saline+L-400 and saline+L-800) and isoproterenol (ISO) groups including ISO+vehicle , ISO+L-200, ISO+L-400 and ISO+L-800 mg/kg. Values are mean ± SEM. #; P<0.01 from respective saline+vehicle value and *; P<0.01 as compared to ISO+vehicle group.

**Fig.3:**
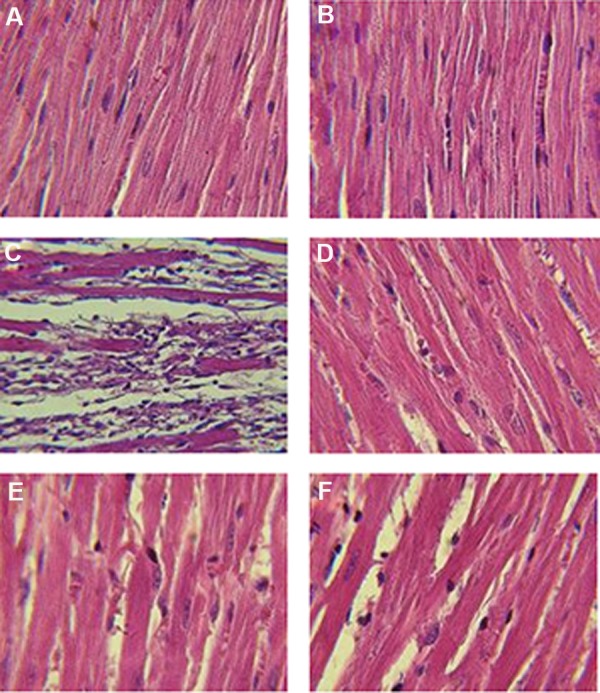
Photomicrograph showing the histopathological changes of heart tissue in normal rats, lavender oil (L) and isoproterenol (ISO) groups including, A. Saline+vehicle, B. Saline+ lavender oil, C. ISO+vehicle, D. ISO+L-200, E. ISO+ L-400, and F. ISO+L-800 mg/kg. H&E (×400 magnification).

**Table 1:** Effect of lavender oil treatment on histopathological alteration in myocardial injury induced by isoproterenol (ISO) in rats


Experimental groups	Myonecrosis	Inflammation	Edema

Normal rat (saline+vehicle)	-	-	-
Normal rat (saline+lavender)	-	-	-
ISO+vehicle	++++	++++	++++
ISO+L-200 mg/kg	+	+	+
ISO+L-400 mg/kg	+	+	+
ISO+L-800 mg/kg	++	++	++


-; Absence of inflammation, edema and myonecrosis, +; Focal areas of inflammation, edema and myonecrosis, ++; Patchy areas of inflammation, edema and myonecrosis, and ++++; Massive areas of inflammation, edema and myonecrosis.

### Effect of pretreatment with various doses of lavender oil on cTnI 

**Fig.4:**
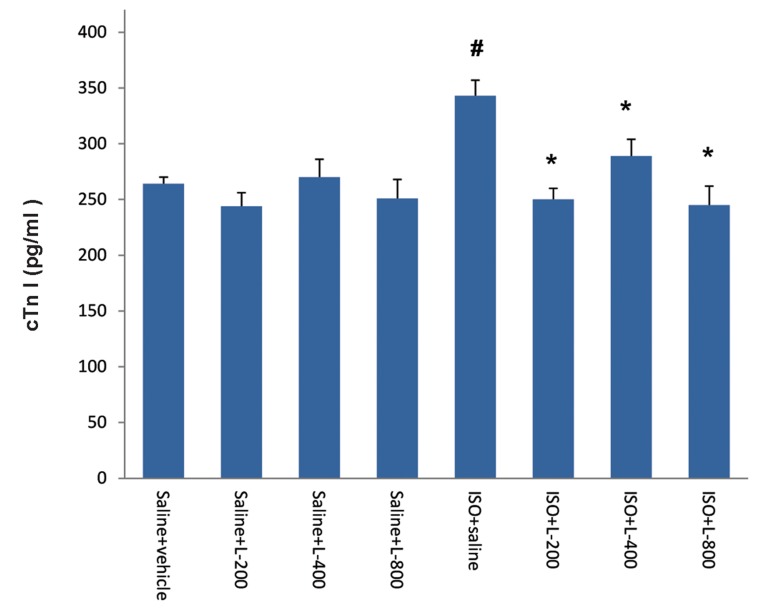
Effect of pretreatment with of lavender oil on plasma cTnI in normal rat (saline vehicle, saline+L-200, saline+L-400 and saline+L-800) and isoproterenol (ISO) groups including ISO+vehicle, ISO+L-200, ISO+L-400 and ISO+L-800 mg/kg. Values are mean ± SEM. #; P<0.01 from respective saline+vehicle value and *; P<0.01 as compared to ISO+vehicle group.

Induction of acute MI by ISO significantly (P≤0.002) increased level of cardiac cTnI in the plasma in comparison with normal control (saline) rats. Pretreatment of rats with lavender oil at all doses resulted in a significant (P≤0.01) decrease in the levels of plasma cTnI, in comparison with the ISO- treated group (Fig.4).

### Effect of pretreatment with lavender oil on heart oxidative stress biomarkers

**Fig.5:**
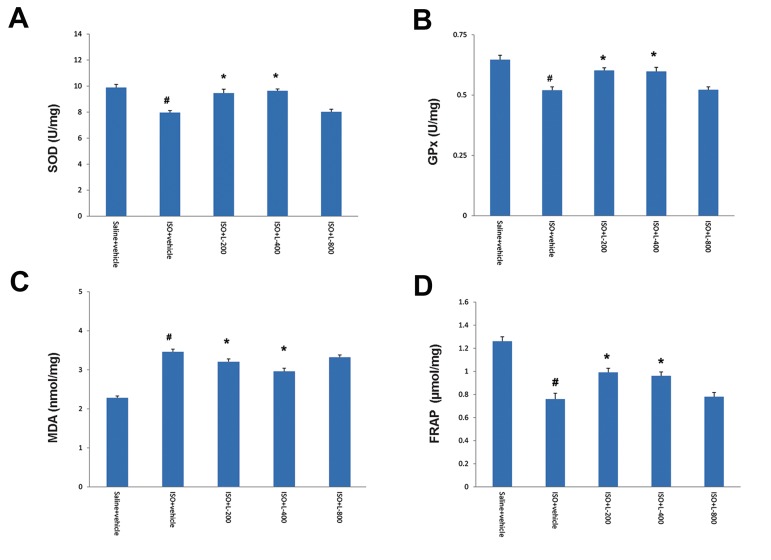
Activity of antioxidant enzymes A. SOD, B. GPx, C. MDA content, and D. FRAP levels in normal rat (saline+vehicle) and isoproterenol (ISO) groups, including ISO+vehicle, ISO+L-200, ISO+L-400 and ISO+L-800 mg/kg. Values are mean ± SEM. SOD; Superoxide dismutase, GPx; Glutathione peroxidase, MDA; Malondialdehyde, #; P<0.01 from respective saline+vehicle value, and *; P<0.01 as compared to ISO+vehicle group.

Induction of acute MI by ISO significantly (P≤0.01) decreased activities of the antioxidant enzymes SOD and GSH-Px and increased levels of MDA in the heart tissue, in comparison with normal rats (Fig.5A-D). Administration of lavender oil at doses of 200 and 400 mg/kg significantly (P<0.01) reduced MDA content as a lipid peroxidation marker (Fig.5C). Pretreatment with lavender oil at doses 200 and 400 mg/kg prevented reduction of antioxidant enzymes (SOD and GSH-Px) (Fig.5A, B) and increased level of FRAP (Fig.5D) in heart tissue, relative to the ISO-treated group.

### Effect of pretreatment with lavender oil on pro-inflammatory cytokine; tumor necrosis factor-alpha 

**Fig.6:**
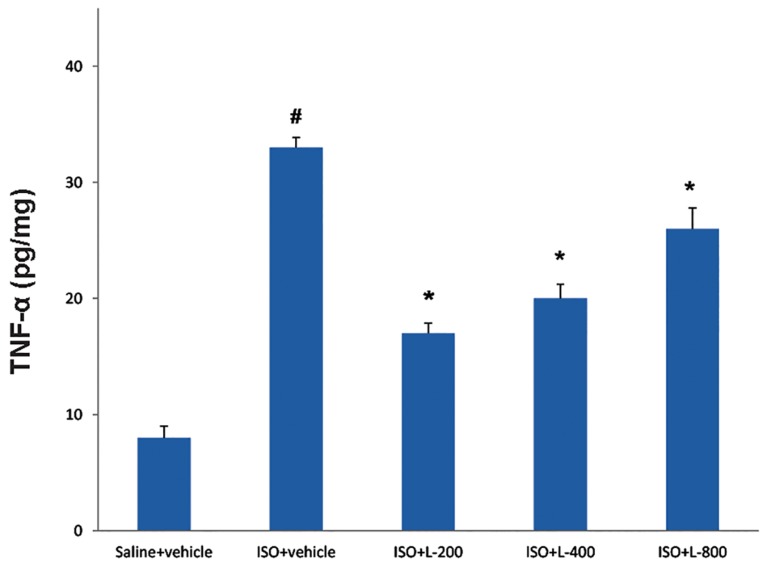
TNF-α levels in normal rat (saline+vehicle) and ISO groups including ISO+vehicle, ISO+L-200, ISO+L-400 and ISO+L-800 mg/kg. Values are mean ± SEM. TNF-α; Tumor necrosis factor-alpha, ISO; Isoproterenol, #; P<0.01 from respective saline+vehicle value, and *; P<0.01 as compared to ISO+vehicle group.

TNF-α levels were significantly increased in ISO-treated group, compared to the vehicle group (P<0.001, Fig.6). Pretreatment with lavender oil at doses 200, 400 and 800 mg/kg significantly reduced TNF-α levels in heart tissue (P<0.001, Fig.6). 

## Discussion

ISO-induced MI is attributed to production of free radicals, which causes cardiac dysfunction, increased lipid peroxidation and depletion of endogenous antioxidants (15, 18). Antioxidant enzymes, such as SOD and GPx, are the first line of cellular defense against oxidative stress damage under pathological conditions (19). The results of our study showed that ISO-induced MI, caused reduction in the endogenous antioxidant enzymes SOD and GPx, increasing lipid peroxidation and diminishing cardiac function.

Pretreatment with lavender oil at doses of 200 and 400 mg/kg, but not 800 mg/kg, significantly reduced myocardial injury, troponin I as well as TNF-α and improved cardiac function, in addition to antioxidant activity (P<0.01). Antioxidant activity of lavender oil in low doses is in agreement with our previous study (13). MDA is a major lipid peroxidation product and a sensitive biomarker of oxidative stress (20). Our result indicated that the content of MDA in the heart was increased after acute MI and that pretreatment with lavender oil restored it to near the baseline level. Increase in the MDA level and/or lipid peroxidation may be due to increased free radical formation and decreased antioxidant enzymes following acute MI. Decreased MDA level in heart following pretreatment with lavender oil may reflect a decrease in the extent of myocardial damage. This finding suggests that at least part of the cardioprotective activity of lavender oil observed in this study is due to the respective antioxidant properties and augmentation of the antioxidant defense system.

Several studies have demonstrated that plants with antioxidant activity have positive effects in prevention and treatment of various diseases induced by oxidative stress (21-24). In fact, different molecular mechanisms protect against diseases induced by oxidative stress, out of which anti-oxidative properties have been determined as one of the most important mechanisms (25- 29). Therefore, lack of antioxidant activity and preventive effect of lavender in higher dose (800 mg/kg) on MI seems to be surprising. Under certain circumstances antioxidants may act as pro-oxidant and promote oxidation of other compounds, inducing tissue damage (30, 31). Production of superoxide anion and lipid peroxidation is elevated with increasing concentrations of flavonoid antioxidants (32).

Furthermore, pro-oxidant compounds are able to induce DNA strand breakage, which has been attributed to hydroxyl radical formation of flavonoids. In this regard gossypol, quercetin and myricetin powerfully inhibited iron-induced lipid peroxidation in rat liver microsomes, at low micromolar concentrations (IC_50_=1.5 µM). However, all three compounds at 100 µM concentration increased the formation of hydroxyl radical up to eight-fold (33). Moreover, in human leucocytes, quercetin at doses of 1-50 µM reduced the levels of oxidative DNA damage; however, at the high dose of 100 µM damaging level was increased. Paradoxical activity of antioxidants has been previously demonstrated by other antioxidant compounds (34, 35). Therefore, lack of cardioprotective effect of lavender at 800 mg/ kg dose might be due pro-oxidation.

Increased production of pro-inflammatory cytokines such as TNF-α and activation of oxidative stress lead to apoptosis and impaired contractile performance of the heart (36). Therefore, suppressing TNF-α and oxidative stress cascade could preserve myocardial function. This study demonstrated that treatment of lavender oil resulted in a significant reduction in the level of TNF-α in heart tissue. These findings are in consistence with our and other studies, which have shown that lavender oil has antioxidant activity (13) and can suppress pro-inflammatory cytokines in lung tissue (37). Therefore, cardioprotection effects of lavender oil observed in this study may be related to inhibition of TNF-α production and oxidative stress damage.

Cardiac troponin I is a sensitive biomarker for diagnosis of acute MI and detection of MI (38). Serum cTnI levels associate with the degree of histological cardiac damage following MI (39). In our study, induction of acute MI by ISO significantly increased the plasma level of cTnI, as a result of leakage from the damaged heart tissues into the blood. Pretreatment with lavender oil restored cTnI to near-normal levels. This effect may have been due to reduction of the extended damage in the myocardium by lavender oil treatment.

Another finding of our study is that dp/dt maximum (contraction velocity), dp/dt minimum (relaxation velocity) and LVEDP, as markers of left ventricular function, were significantly decreased in ISO-treated rats, as previously reported (15). Our study showed that pretreatment with lavender oil considerably improved left ventricular systolic dysfunction (LV dp/dt maximum and LVSP) and mean arterial blood pressure, as well as left ventricular diastolic dysfunction (LV dp/dt minimum and LVEDP). This finding is important because it shows that heart ischemic injury was reduced along with improvement of left ventricular cardiac function.

Histopathological scores confirmed the extent of cardiac damage in experimental models of MI (15). An increase in this parameter reflects the extent of myocardial damage (39). Our study indicated that induction of acute MI by ISO remarkably enhanced histopathological scores as well as necrosis of muscle fibers with inflammatory cell infiltration, edema and fragmentation of muscle fibers. Pretreatment with lavender oil markedly reduced histopathological scores as well as preserving myocardial fiber structure and architecture. The preserved morphology of cardiac myofibers lends additional confirmation of the cytoprotective effect of lavender oil.

## Conclusion

Our findings exhibited that lavender has cardioprotective effect through reducing synthesis of TNF-α, augmentation of the antioxidant defense mechanism and inhibition of oxidative stress. We suggest that lavender might be helpful in prevention or attenuation of heart injury in patients with high risk of myocardial infarction and/or ischemic heart disease. 
